# The Distribution of Phytoplasmas in South and East Asia: An Emerging Threat to Grapevine Cultivation

**DOI:** 10.3389/fpls.2019.01108

**Published:** 2019-09-11

**Authors:** Roberto Pierro, Teodoro Semeraro, Andrea Luvisi, Harsh Garg, Marzia Vergine, Luigi De Bellis, Harsimran K. Gill

**Affiliations:** ^1^Department of Agriculture, Food and Environment, University of Pisa, Pisa, Italy; ^2^Department of Biological and Environmental Sciences and Technologies, University of Salento, Lecce, Italy; ^3^School of Life and Environmental Sciences, University of Sydney, Sydney, NSW, Australia; ^4^Crop Science Division, Baern U.S., Ithaca, NY, United States

**Keywords:** phytoplasma, grapevine, grapevine yellows, flavescence dorée, bois noir

## Abstract

Grapevine is largely cultivated in several parts of the world, and a spurt in its cultivation has occurred in the last two decades in grapevine cultivated areas of South and East Asia, mainly in China, India, Japan, Korea, Thailand, and Indonesia. Grapevine yellows (GY) represent one of the most important diseases in viticultural areas of the world, and they have been assigned to five different groups: aster yellows [AY (16SrI)], peanut witches’ broom [PnWB (16SrII)], X-disease (16SrIII), elm yellows [EY (16SrV)], and Stolbur (16SrXII). This study provides a comprehensive overview of the presence of phytoplasma strains and their vectors associated with GY complex, and their potential impact on viticulture of the South and East Asia. In general, both AY and EY were reported on several herbaceous plants and/or cultivated plants in South and East Asia, along with its vectors that were largely reported in China and sporadically in Japan. Interestingly, AY and EY are yet not found in South and East Asia grapevine regions; however, their presence on different plant species suggests the potential spread of the pathogens that may occur in grapevine regions in the near future. Additionally, a few reports also suggest the presence of Stolbur group in Asian countries, along with one study that found a Stolbur-related strain in China on *Vitis vinifera*. Similarly, PnWB was also frequently reported in India and China on several plant species, but not in grapes. Conversely, sporadic detections of phytoplasma strains related to X-disease in Thailand, South Korea, and China indicate that their potential influence in viticulture is rather negligible. Our review suggests that monitoring and control strategies against GY are essential in order to prevent epidemic phytoplasma spread, especially in vine-allocated areas in Asia.

## Viticulture in South and East Asia and Pest Globalization

**Figure 1:**
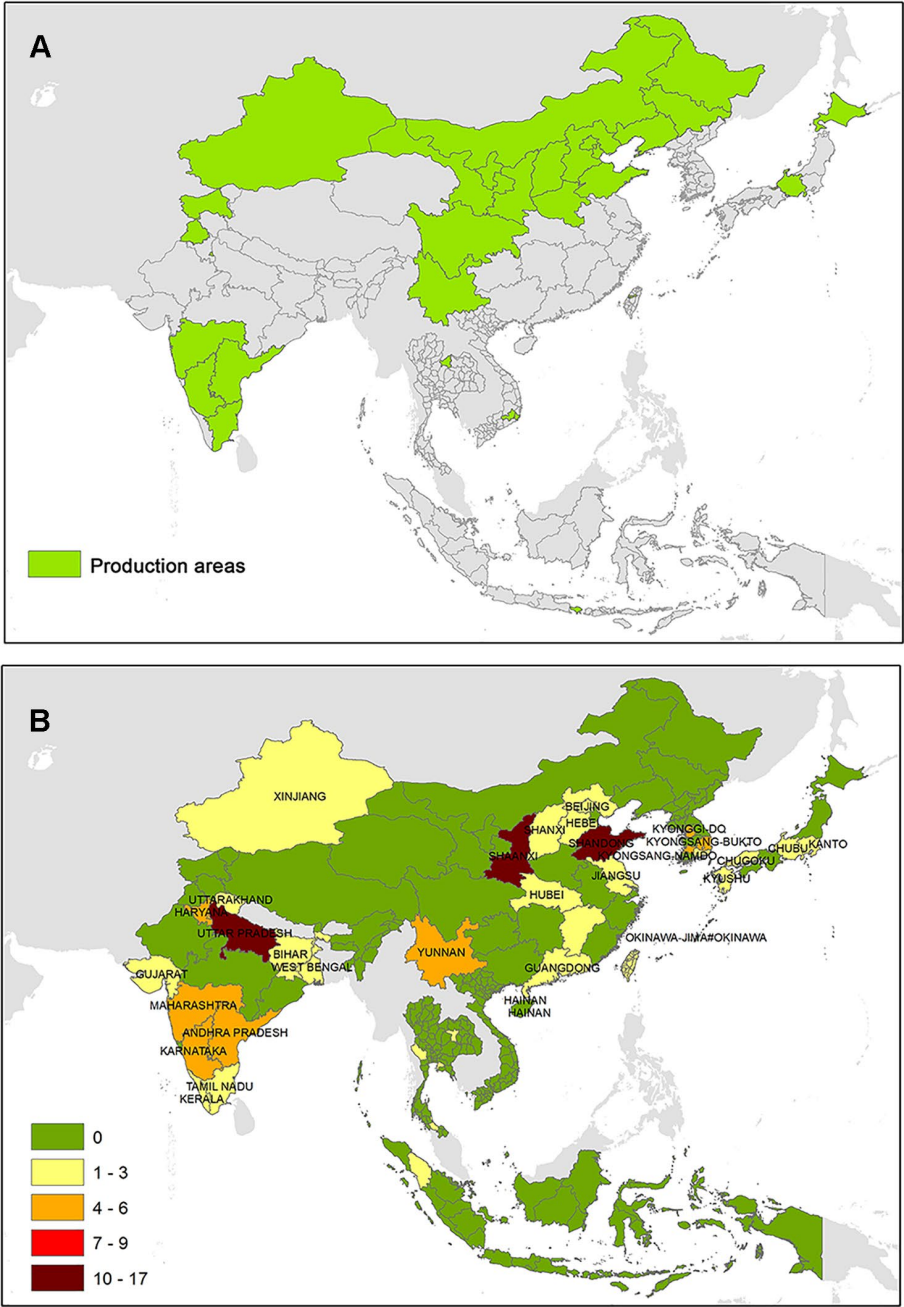
**(A)** Major vine-growing areas South and East Asia and **(B)** evidences of phytoplasma associated with *Vitis vinifera*.

Global trading of plants and plant produce expedite the international pest movement. The introduced pests in an area lead to major economic consequences for regional plant production, and it should be avoided at any cost (Suffert et al., 2018). Risks related to pest introduction are widespread, and control may be particularly warning when novel cultivations are introduced or expanded. Over the last 30 years, the grape industry encountered peak levels of economic growth among the other agricultural commodities, and grape and wine production became the multibillion-dollar global venture. This growth is linked with many factors such as public awareness of health benefits of grapes as an antioxidant, increase in international trade, changing policies, and improved global incomes (Danne et al., 2018). Grapevine is a high-value perennial crop (Suffert et al., 2018) that caters for diverse markets such as processed grapes, which are dried into raisins or pressed into wine or grape juice, and table grapes for fresh consumption (Danne et al., 2018). Grapevine is largely cultivated in several parts of the world, and in the last two decades, its cultivation has largely been extended to South and East Asian countries in vine-allocated areas mainly located in China, India, Japan, Korea, Thailand, and Indonesia (Anderson and Wittwer, 2015) (**Figure 1A**). Currently, China represents one of the most important wine producers in the world (Li and Bardají, 2017), thanks to several years of winemaking and availability of wide geographic sizes for vineyard plantation in distinct topographic situations. In 2017, China and Hong Kong exported the highest dollar-value worth of fresh grapes along with other 13 countries worldwide: $735.2 million (8.8% of total exported grapes) and $363.2 million (4.4%), respectively. China (up 173.7%) was documented to be the fastest-growing grapes exporters since 2013 along with Uzbekistan (up 244.1%), Mexico (up 63.7%), and Australia (up 49.4%) (http://www.worldstopexports.com/grapes-exports-by-country/).

More than 1/10th of all pests have already been outstretched to more than half of the countries that grow their host plants or crops. If this scenario continues, then by the middle of the century, many specific-crop-producing countries will be fully saturated with pests. The global dispersal of some pests has been speedy, but pest aggregation remains strongly regional and follows the host plants’ distribution (Bebber et al., 2014). Thus, the presence of phytopathogens that are extremely difficult to control such as phytoplasmas in vine-growing areas of South and East Asia should not be disregarded.

## How Grapevine Yellows Are Becoming a Global Problem

Several hundreds of plant diseases, including the grapevine yellows (GY), have been associated with phytoplasma-associated complex. Phytoplasma classification adopted the highly conserved rRNA gene sequences for phytoplasma classification in ribosomal groups. The molecular approach showed that phytoplasmas constitute a large monophyletic group within the class Mollicutes (Lee et al., 1992; Lee et al., 1998; Quaglino et al., 2009) and allowed the designation of a new taxon named “*Candidatus* Phytoplasma” (IRPCM, 2004). Currently, 33 groups were identified, and each of them has been proposed to represent in at least one species. Several groups and subgroups were officially designed as “species” under the provisional status “*Candidatus*,” while other provisional “species” have not been formally described yet (i.e., “*Candidatus* Phytoplasma vitis”) (IRPCM, 2004).

GY represent one of the most important phytoplasma diseases in all major viticultural areas of Europe (Maixner et al., 1995), the USA (Davis et al., 1998), and Asian countries such as China (Cai et al., 2016; Jiang et al., 2009), India (Yadav et al., 2016; Tripathi et al., 2017), Korea (Chung and Kim, 2005), Japan (Takinami et al., 2013), and Thailand (Sarindu and Clark, 1993).

On the basis of the classification system, GY phytoplasmas have been assigned to five different ribosomal groups: aster yellows [AY (16SrI)], peanut witches’ broom [PnWB (16SrII)], X-disease (16SrIII), elm yellows [EY (16SrV)], and Stolbur (16SrXII) (Bianco et al., 1993; Davis et al., 1993; Maixner et al., 1995).

The EY phytoplasma (16SrV) group consists of diverse phytoplasma strains that cause a decline in American elms in several plant species worldwide. On the basis of phylogenetic analysis of 16S rDNA sequences, within the EY group, six subgroups were identified (16SrV-A to 16SrV-F), associated with different outbreak severities and geographical distributions (Bertaccini et al., 2014).

Within this group, phytoplasma associated with “Flavescence dorée” (16SrV-C and 16SrV-D) (FD) in grapevine surely represents one of the most important GY (IRPCM, 2004) occurring in all major vine-growing areas of Euro-Mediterranean countries (where it is classified as quarantine pest), Chile, and Asia (Choueiri et al., 2002; Gajardo et al., 2009; Belli et al., 2010; Duduk et al., 2010; Salem et al., 2013; Mirchenari et al., 2015), due to the transmission activity of the ampelophagous leafhopper *Scaphoideus titanus*.

Within the Stolbur group, eight subgroups have been described (16SrXII-A to 16SrXII-H) (Quaglino et al., 2009; Bertaccini et al., 2014). The presumptive causal agent of Bois noir (BN) was identified in the phytoplasma “*Ca. P. solani*” to belong to the subgroup 16SrXII-A (Quaglino et al., 2013). Due to the erratic feeding activity of the polyphagous planthopper *Hyalesthes obsoletus*, it is widespread in several Euro-Mediterranean countries, China, and Chile (Pierro et al., 2018), where it caused severe crop losses in almost all *Vitis vinifera* varieties.

On the basis of their epidemiology, FD cycle is quite different from that of BN. In particular, FD causal agent is vectored from vine to vine by the ampelophagous leafhopper *S. titanus*, while BN etiological agent is mainly transmitted by the polyphagous planthopper *H. obsoletus*, strongly dangerous for grape production.

One of the most genetically variable phytoplasma strains are those belonging to the peanut witches’ broom (16SrII), which likely originated from Asia. Within this group, 23 subgroups, considered as relatives of “*Ca. P. aurantifolia*” or “*Ca. P. australasiae*” (IRPCM, 2004) and found in association with numerous plant diseases, have been classified (Yang et al., 2017).

GY can be associated also with phytoplasma strain belonging to the AY subgroups 16SrI-A and 16SrI-B (“*Ca. P. asteris*”) (Lee et al., 2004) and X-disease (16SrIII) groups (“*Ca. P. pruni*”) (Davis et al., 2013), previously identified in diseased grapevines in the USA and northern Italy (Davis et al., 1998; Bertaccini et al., 2014).

Outbreak of GY epidemics could be a concrete risk in the vineyard agro-ecosystems in all viticultural areas with relative negative economic impact. Monitoring and control strategies against GY, especially for FD, are essential in order to prevent epidemic phytoplasma spread, both in historical vine-growing areas in European countries and in more recent allocated vine areas in the USA and Asia. Indeed, the FD risk for the EU territory was recently analyzed with a quantitative approach, for example, evaluating the FD impact reduction due to hot water treatment for planting material produced in infected areas or evaluating the impact of eradication and containment measures (Jeger et al., 2016). This work is an outlook about GY conditions in vineyard agro-ecosystems located in South and East Asia. Our literature survey included articles, books, and conference papers (retrieved from Scopus database, ResearchGate, and Google Scholar) published in the last two decades (1998–2018) or over than some historical reports.

## China and Taiwan

The Chinese wine industry has rapidly developed since the 1950s, with more than one billion liters of wine production in the last years. Since the last two decades, Taiwan, the island province of the Republic of China, is able to develop a new economy based on their vine production. In northwestern China (NW), the main vine-growing areas are the autonomous regions of Xinjiang and Ningxia and the provinces of Shaanxi and Gansu. In north China (N), besides Beijing and Tianjin Municipality, grape is cultivated in Hebei, Shanxi, and in the autonomous region of Inner Mongolia. Each province of northeast China (NE) (Liaoning, Jilin, and Heilongjiang) is involved in viticulture, while the province of Henan is the most important vine area of south central China (SC). In east China (E), vineyards are farmed in Shandong, whereas the vines are widespread in the province of Yunnan and Sichuan in the southwest of China (SW) (Puckette, 2012; Li and Bardají, 2017). The Cabernet Sauvignon is the most widely planted wine grape in China with more than 20,000 ha, followed by Chardonnay, Cabernet Franc, Syrah, and Pinot (Li and Bardají, 2017).

**Figure 2:**
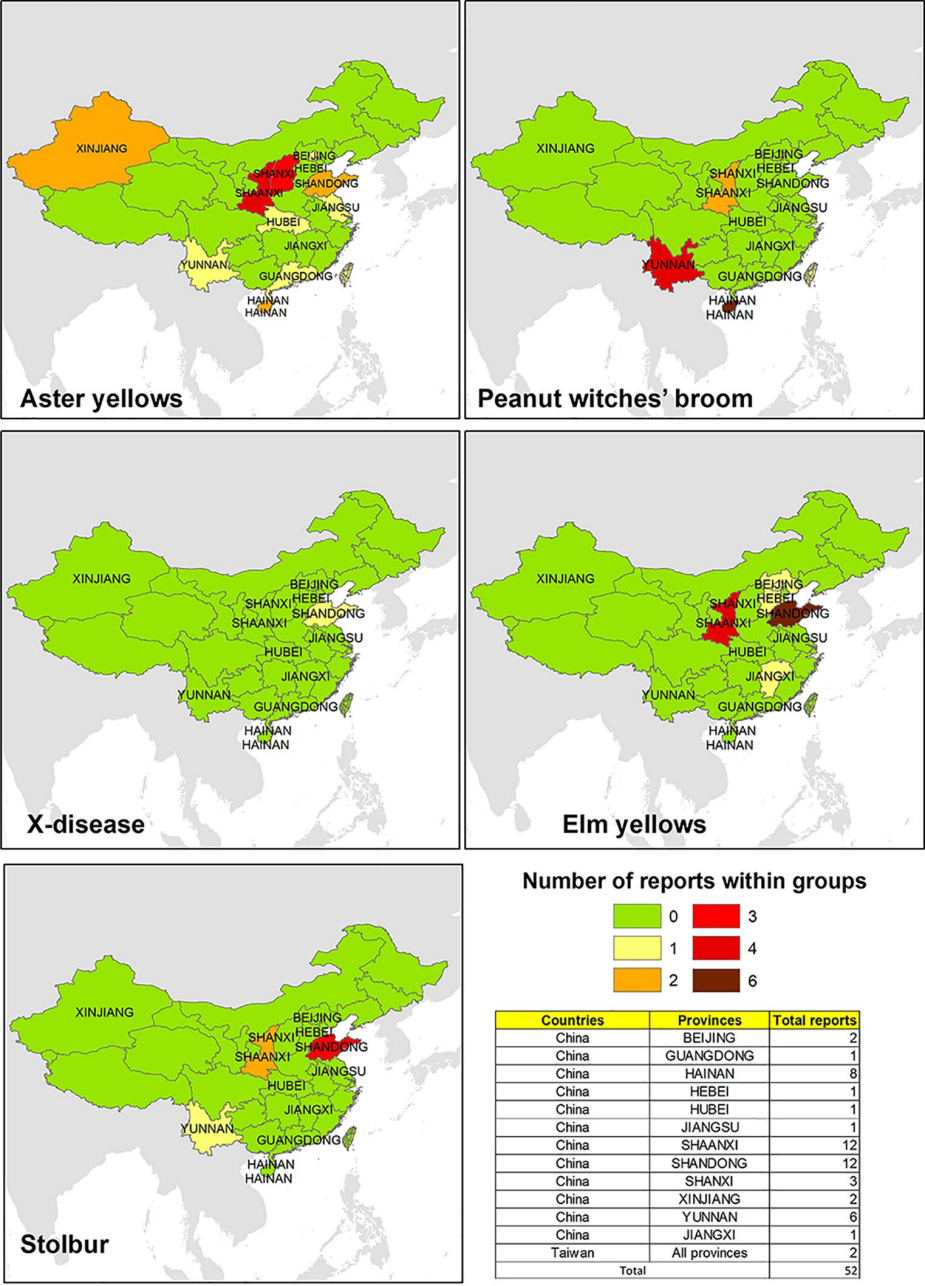
Evidences of phytoplasma 16Sr ribosomal group reported in different areas of China and Taiwan.

Surveys on phytoplasma strains potentially infecting *V. vinifera* in China are mainly focused in Shandong, Shaanxi, Yunnan, and Shanxi, while some evidences about the pathogens are also retrieved from the other six provinces (**Figure 2**). As shown in **Figure 2**, evidences of AY (16SrI) phytoplasmas are the most common, which were repeatedly found in many areas of China (except for the northeast), for example, in Xinjang, Shanxi, and Shandong, where the pathogen was detected in wooded plants such as *Prunus* (Zhang et al., 2013a; Wang et al., 2018) or *Citrus* (Chen et al., 2009) and in economically important crops such as *Triticum aestivum* (Wu et al., 2010). EY phytoplasma-related strains were mainly identified in the east and north areas, such as in Shandong and Hebei, which harbor several plant hosts (Yu et al., 2012; Li et al., 2014). Conversely, the presence of phytoplasma strains associated with the PnWB was largely reported in SC and SW in important crops such as *Lycopersicum esculentum* (Xu et al., 2013) and *Brassica oleraceae* (Cai et al., 2016). Phytoplasmas related to the Stolbur group were reported in different vine-growing areas in China, and they were identified in the Shaanxi autonomous region (NW) in *V. vinifera* plants (Duduk et al., 2010), suggesting the presence of phytoplasma strains associated with BN.

A few studies also suggest the presence of phytoplasma associated with AY and PnWB in Taiwan on different cultivated crops as *Hibiscus sabdariffa* and *Sesamum indicum* (Tseng et al., 2014a; Tseng et al., 2014b).

Taking into account that the phytoplasmas are mainly spread by insect vectors or *via* vegetative propagation (grafting or cuttings) and *V. vinifera* can be a natural host for several phytoplasma strains, the risk of outbreak of phytoplasmas diseases in viticultural areas of China and Taiwan in the future could be facilitated by vegetative propagation, import of infected plant material, and discovery of new or already known insect vectors that are able to spread phytoplasma strains in grapevines. This opens up new challenges in the Chinese and Taiwanese agro-ecosystem vineyards.

## India

**Figure 3:**
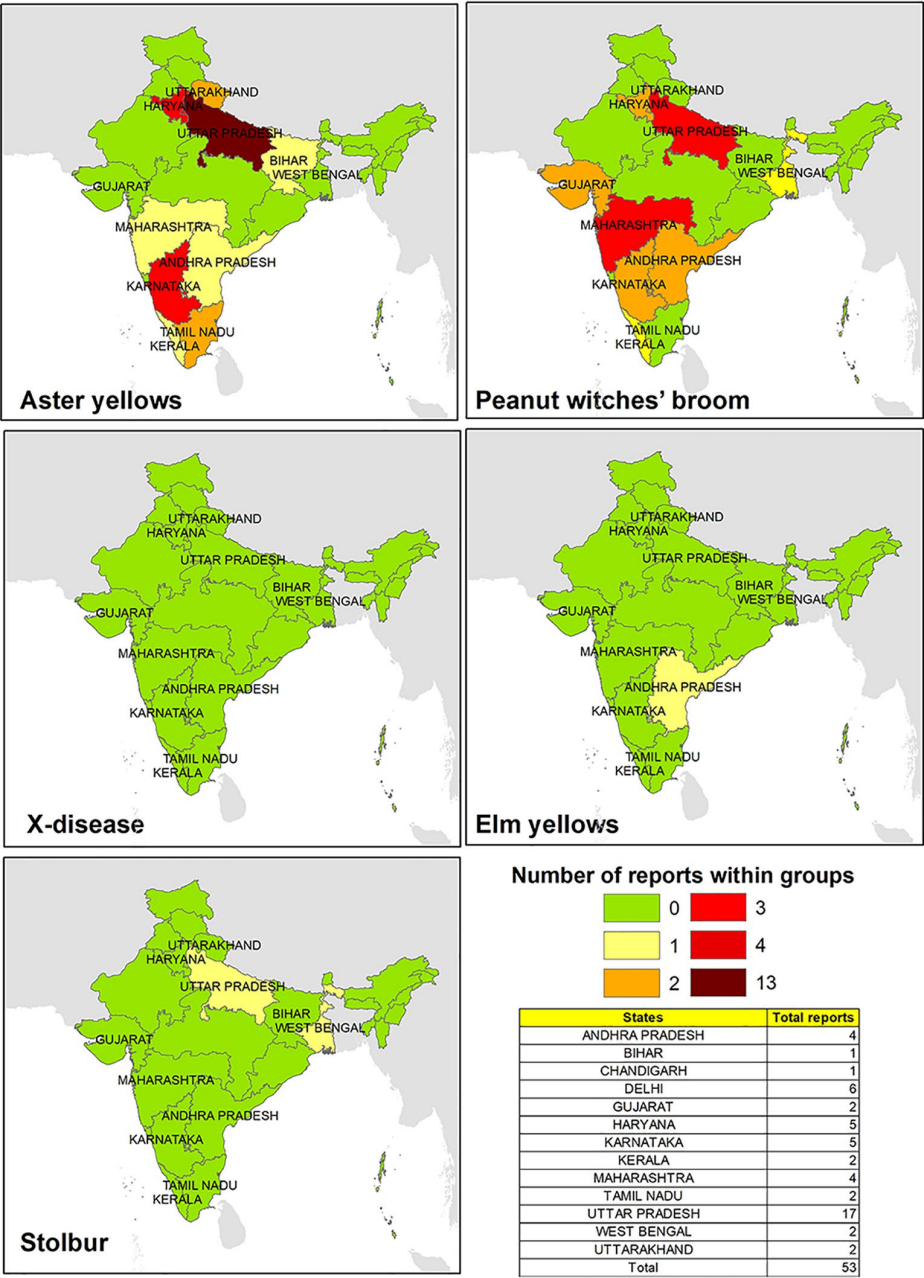
Evidences of phytoplasma 16Sr ribosomal group reported in different parts of India.

The main wine-producing areas in India are located in the northwestern parts of Punjab and Kashmir, central southern areas of Maharashtra, Goa, Karnataka, Andhra Pradesh, and Tamil Nadu states. The presence of phytoplasma strains belonging to ribosomal groups potential infecting *V. vinifera* plants in India was mainly related to AY and PnWB (**Figure 3**). In particular, phytoplasma associated with the AY group was reported to be prevalent spread in the northern part of the country on several and economically important crops such as *Capsicum annuum* and *Gossypium hirsutum* (Khan and Raj, 2006; Kumar et al., 2010), while phytoplasma related to the PnWB group has been equally reported in the southern and central southern areas of the country.

The total number of reports that has been reported for different groups of phytoplasmas is rather limited, even though India is one of the most important agricultural economic countries in the Middle East. It is possible that the practices of pesticides and/or removing phytoplasma-infected plants (Firrao et al., 2007; Bertaccini et al., 2014) could have limited the disease manifestation or outbreaks in Indian viticulture area, and that would have possibly affected its further investigations.

## South Korea and Japan

The main important vine-growing areas are located in the South Korea, where vine production grew from 1995, and it is currently increasing. The main vine-growing areas in the South Korea are mainly localized in the central part of the country. In particular, the Kyongbuk province represents the largest grapes area (46%), followed by Chungbuk (15%) and Chungnam and Kyonggi (10% for both) (www.fao.org). Conversely, wine production in Japan has progressed in all the four main islands from the north to the south. The major wine-producing regions are Yamagata, Yamanashi, and Nagano prefectures in the northern and central parts and Okayama and Fukuoka prefectures in the southern part.

**Figure 4:**
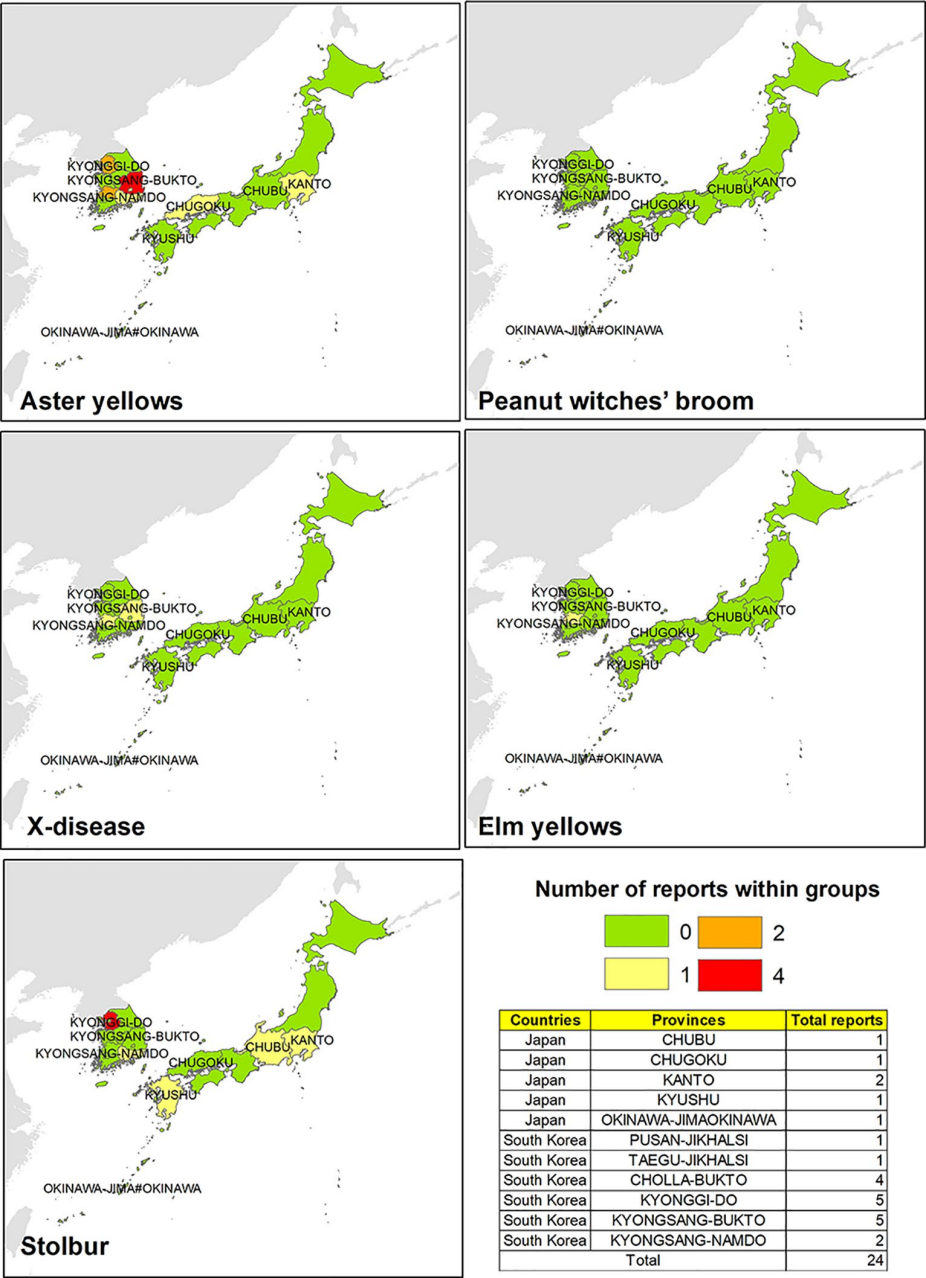
Evidences of phytoplasma 16Sr ribosomal group reported in different South Korean and Japanese areas.

Researches focused on potential phytoplasma strains infecting *V. vinifera* in such Asiatic areas revealed that phytoplasma diseases are more diffused in South Korea than in Japan. In particular, AY is the most spread phytoplasma ribosomal group in the central and southern parts of South Korea, followed by a few records of phytoplasmas associated with the Stolbur group (three reports in the north and one in the south) (**Figure 4**). The only report of phytoplasma identification related to EY group was recorded in *Hovenia dulcis* by Kamala-Kannan et al. (2011).

Sporadic detection of phytoplasma strains associated with Stolbur and AY groups were reported in Japan, revealing that such diseases are mainly localized in Kanto, Chubu, Kyushu, and Chugoku areas. Phytoplasma strains belonging to the Stolbur group were identified only in *Hydrangea* spp. hosts (Sawayanagi et al., 1999), and the presence of phytoplasma disease in chrysanthemum (*Dendranthema grandiflorum*) caused by “*Ca. P. aurantifolia*” was also unveiled by the 16S rDNA sequencing (Takashi et al., 2007).

## Thailand and Indonesia

The grape industry in Thailand has been a success since 1956. The initial commercial wine-producing areas were located in the central plain region at Nakhon Pathom, Ratchaburi, Samut Sakhon, and Samut Songkhram provinces, close to Bangkok. At present, the grape industry has expanded to the northern, northeastern, central, and western regions (Nilnond, 2001). However, in Indonesia, grapevine cultivation is mainly located in east Java only (Bali, Probolinggo, and Malang), where its wine production has remained limited.

**Figure 5:**
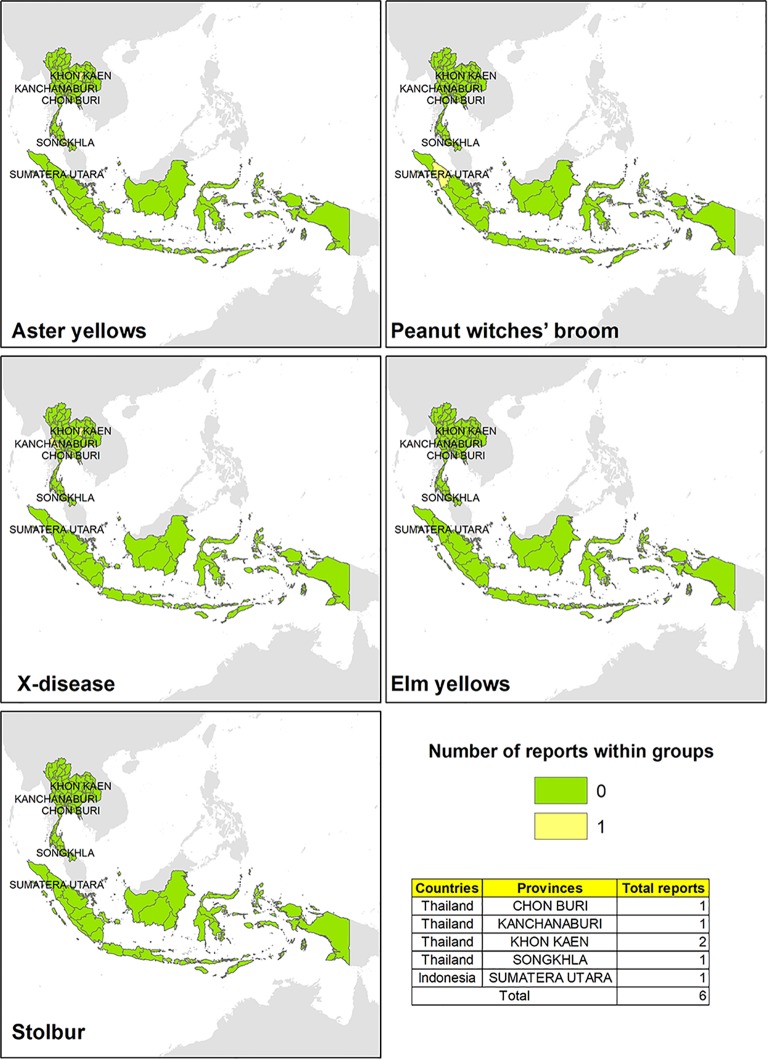
Evidences of phytoplasma 16Sr ribosomal group reported in different areas in Thailand and Indonesia.

A few evidences reporting the presence of phytoplasma ribosomal groups have been found in Thailand and in Indonesia (**Figure 5**). In Thailand, phytoplasmas associated with AY and X-disease groups were reported on plant hosts such as *Axonopus compressus*, *Cynodon dactylon*, *Saccharum officinarum*, *Solanum betaceum*, and *Capsicum* spp. (Sdoodee et al., 1999; Harling et al., 2009; Soufi et al., 2013), while group 16SrII (“*Ca. P. aurantifolia*”) was found on host plants such as chili (*Capsicum* spp.) and tamarillo (*Cyphomandra betacea*) in the west side of the Indonesia (Harling et al., 2009).

## Role of Vectors in Spreading GY: The Asian Perspective

Vector is a carrier that comprises any living organisms, which carries and transmits an infectious pathogen such as bacteria or virus into other living organisms or plants or any other living objects. There have been numerous troublesome invasions by arthropods pests into grape production, which have threatened sustainability. Arthropod pest invasion leads to lower crop yields, higher management costs, and environmental impact (Danne et al., 2018). In most wine grape regions, the transmission of plant pathogens, rather than damage caused by insect feeding, is a serious concern (Charles et al., 2010). GY phytoplasmas are spread by insect vectors, which feed inside the phloem tissues of grapevines.

**Figure 6:**
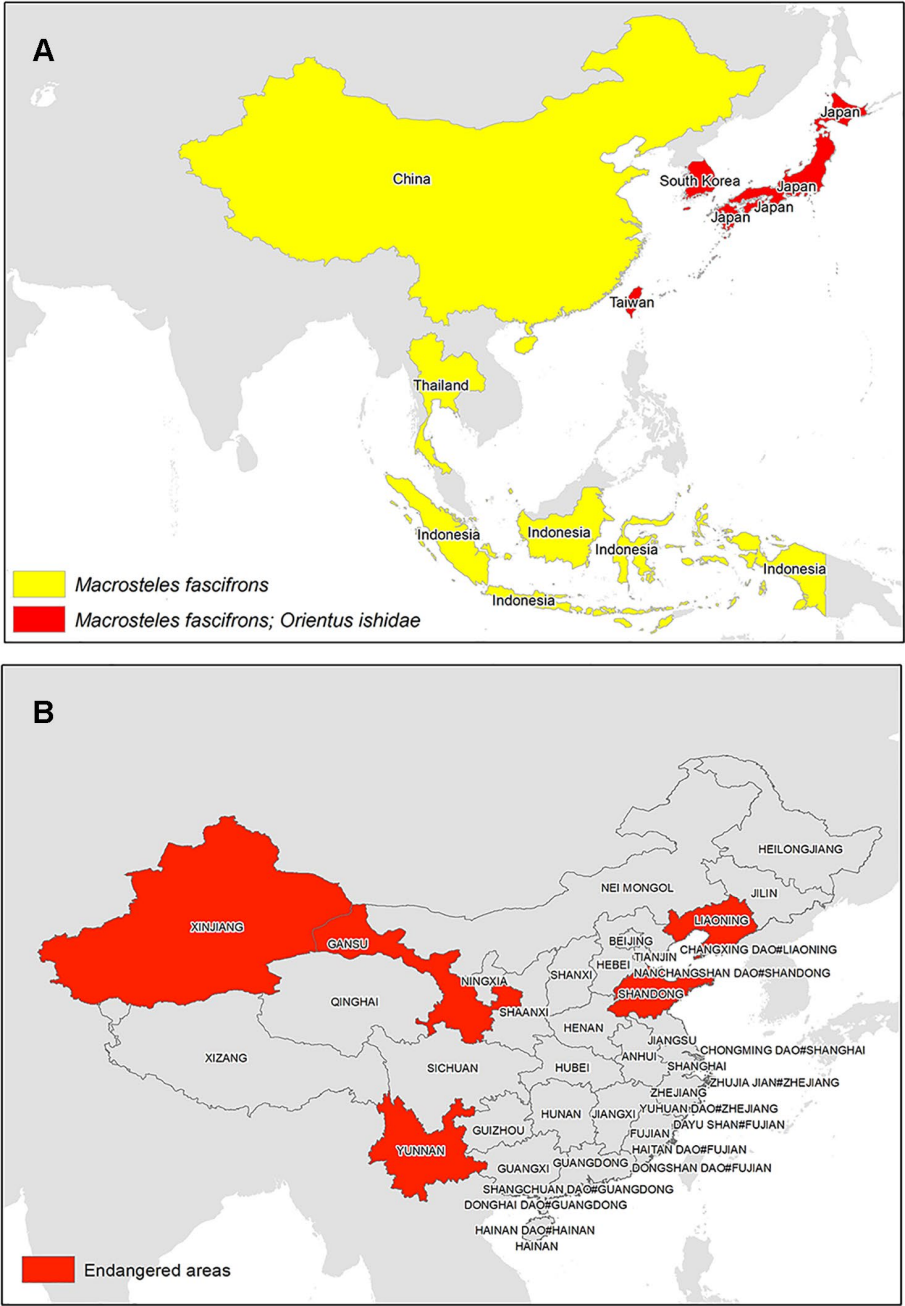
**(A)** Evidences of grapevine phytoplasma vectors reported in South and East Asia and **(B)** Chinese provinces predicted to be potentially endangered owing to *Scaphoideus titanus* (Ge and Wen, 2006).

The success of establishment and spread of grapevine phytoplasmas depends upon the type of the insect vector and its feeding behavior. A vector with high transmission frequency, produces several generations per year, and is polyphagous in nature has a higher potential to spread the disease than do the ones with narrow plant host range and few or only one generation a year, with low transmission frequency and poor growth rate. A number of vectors listed earlier have been reported to spread grapevine phytoplasmas. Though the occurrence of these vector in North America and European regions have been well established, very few studies have been reported that indicate the presence of these vectors in South and East Asia (**Figure 6**). However, in this era of trade and globalization, their spread is imminent, especially due to the ease and ability of these vectors to adapt in new geographic regions.

*Macrosteles fascifrons* is one of the grapevine phytoplasma vectors that cause AY. It is a polyphagous leafhopper and occurs predominantly in grapevine field as reported in Canadian vineyards (Saguez et al., 2014). Few incidences of the vectors are reported in China (Li, 1985; Zhang et al., 2013b; Zhang et al., 2013c) and Japan (Shiomi and Sugiura, 1983), which indicates the potential of its further spread to other regions. Another GY phytoplasma vector reported in Japan on apple tree is *Orientus ishidae* (Ishihara, 1968; Guglielmino, 2005). It is a polyphagous vector and is associated with the “FD” 16SrV phytoplasmas (Lessio et al., 2016). Interestingly, *O. ishidae* is considered to be an Asian species, whose introduction in Europe is supposed to have occurred recently in 1998 (Guglielmino, 2005; Lessio et al., 2016). The vector is now widespread in Europe (Lessio et al., 2016), which suggests its potential to adapt and spread to new geographic locations. Moreover, phytoplasmas belonging to 16SrVII-A (“*Ca. P. fraxini*”-related) and 16SrVI (“*Ca. P. trifolii*”-related) subgroups were also identified in Italian grapevine and in *O. ishidae* retrieved in the same areas (Zambon et al., 2018), increasing the role of this insect as vine threat.

*S. titanus* is vector of FD that causes 16SrV phytoplasmas. Though the vector is monophagous in nature, it has been classified as a quarantine pest in European region. The disease is still spreading in Europe, and mandatory insecticide sprays have been recommended to control the pathogen (Chuche and Thiery, 2014). *S. titanus* has not been reported in Asia, even though one of the studies in China predicts that it has potential to establish in middle and eastern China (Ge and Wen, 2006). The results show that the vast area of middle and eastern China (101–124°E, 23–42°N) is the potential distribution region of *S. titanus*. Besides, the vector can also survive in the vicinity of Shihezi, in the Xinjiang Uygur Autonomous Region, and in the middle mountain area of Taiwan Province. Further analysis revealed that the potential distribution region and best habitat of FD phytoplasma are identical with those of *S. titanus*. Among the 10 main viticulture regions of China, the Shandong and Liaodong peninsula of the Bohai Gulf viticulture region, the Yunnan plateau viticulture region, and part of the Gansu viticulture region are the most endangered areas by FD and *S. titanus*. As consequences of global warming, the potential distribution regions of FD and *S. titanus* in China will shift north within the coming 50 years. Meanwhile, their potential distribution within the Bohai Gulf viticulture region will expand remarkably (Ge and Wen, 2006). As human activities have been attributed to the long-distance spread of the vector (Chuche and Thiery, 2014), there is a high potential that the vector may spread in Asia and can cause economic losses.

It is also important to consider that phytoplasmas of the taxonomic group 16SrV-C can occasionally transmit to grapevine from *Alnus glutinosa* by the leafhopper *Oncopsis alni* or by the planthopper *Dictyophara europaea* from *Clematis vitalba*, as reviewed in European Food Safety Authority (EFSA) (2014) Plant Health Panel, currently without the possibility of FD outbreaks without the presence of *S. titanus*.

Evidence for the presence of other vectors of GY (i.e., *Fulgoromorpha* spp., *H. obsoletus*, *Reptalus panzeri*, and *Euscelis incisus*) in South and East Asia is lacking, even though they are widely reported in Europe. Further studies that evaluate vector populations in different grapevine-growing areas in Asia may determine the presence of these vector and/or new potential vectors involved in the GY epidemiological patterns in such areas. However, it is important to exchange the certified seeds that are free of diseases and vector across the borders, to limit the distribution of GY vectors.

This study revealed the presence of different phytoplasma groups associated with GY in Asia and their prevalence in diverse geographic regions. These evidences open new interesting perspectives on phytoplasma adaptability in new environments and the potential development of new epidemiological patterns. All data could open useful investigation on phytoplasma biological cycles and strategies for epidemic control in the near future.

## Concluding Remarks

Phytoplasmas are associated with diseases in several hundreds of wild and cultivated plant species, including economically important crops such as grapevine. Currently, GY represent one of the most important diseases in several viticultural areas in Europe, America, Australia, and Africa (Bertaccini et al., 2014). Moreover, in the last decades, an extensive increase in vine-growing areas has been reported in China, India, Japan, Korea, Thailand, and Indonesia. This has further increased the risks related to the introduction of grapevine phytoplasma diseases and their potential spread through the commercial exchanges of infected propagation material, which can threaten the vineyard ecosystems and grape industry in Asian countries.

This study provided a comprehensive overview concerning the presence of phytoplasma strains associated with GY complex and their potential influence in viticulture of the South and East Asia (**Figure 1B**). Phytoplasma strains associated with AY were the most reported phytoplasma disease over all examined Asian countries. In particular, AY were largely detected in several herbaceous and woody plants in north of India, several areas of China, and central and southern areas of South Korea. AY is related to the taxon “*Ca. P. asteris*,” and it is transmitted by more than 20 leafhopper species (Bahar et al., 2018). Among these, *M. fascifrons* is considered one of the AY vectors, and, based on our data, its presence was largely reported in China and sporadically detected in Japan. The presence of AY disease as well as its vector suggests that the pathogen has a potential to spread further and hence poses a serious threat to existing and new vine cultivated areas of Asiatic regions.

In China, the presence of phytoplasma strains belonging to EY group was also reported in several cultivated plants, and no *S. titanus* was identified in that area. Nevertheless, considering the high probability of *S. titanus* diffusion in middle and eastern China (Ge and Wen, 2006; Chuche and Thiery, 2014) and the wide areas allocated to the vine cultivation, there is possibility that the phytoplasma related to the EY group may have significant impact in the grape industry in Asia, if vigilant control measure that limits its spread will not be imposed. Monitoring and preventing activities should particularly be aimed to FD phytoplasma, which is widespread in Europe and represents the most dangerous grapevine pathogen and is also classified as a quarantine pest.

Reports related to the identification of phytoplasma strains associated with Stolbur group were also evaluated. In particular, there are few findings about “*Ca. P. solani*” strains identification over Asian countries, and only one study conducted by Duduk et al. (2010) in China reported the occurrence of strains related to Stolbur group in *V. vinifera*, which is related to BN disease. Up to now, the main insect vectors of BN have not been reported in any Asian countries, but the presence of BN-infected vines in this Asian area could indicate the presence of BN phytoplasma insect vectors (not yet identified) or the possible role of new putative insect vectors involved in the BN epidemiological pathways in Asia. In this issue, further investigations to identify new BN-infected vines and insect vectors involved in the disease epidemiology are essential tools in order to prevent BN spread on a wider geographical scale.

There have been reports about the presence of phytoplasma associated with PnWB in India and China, where it negatively influenced the production of economically important crops (papaya, sesame, legume, citrus, etc.). In this case as well, the potential transmission of such phytoplasma strains to grapevines is a threat for vineyard management and grape production. In contrast, sporadic detections of phytoplasma strains related to X-disease in Thailand, South Korea, and China, suggest that its potential influence on viticulture is rather negligible.

Overall, monitoring of pests and trading of certified plants are the most crucial steps to prevent the GY spread. It is also important to constantly examine both the asymptomatic and symptomatic plants, alternative host plants in the vineyards and in the surrounding areas, and insect vectors, as new variants of the phytoplasma are constantly emerging. For example, Zambon et al. (2018) detected new strains of phytoplasmas from the main viticulture areas of the northern Italy from both grapevine and insect vector, indicating the potential of the pathogen to evolve, spread, and further cause damage to the new vineyard areas. Indeed, disease prevention and control should also consider the potential role of phytoplasma strains belonging to the 16SrVI and 16SVII-A associated with emerging diseases in grapevine in Italy, China, Syria, and Iran (Zambon et al., 2018), opening new possible epidemiological patterns in vineyard ecosystems. In this contest, pest monitoring is a prerequisite for effective decision making in integrated pest management programs. Markets for agri-food products are changing in developing countries at a pace that is unequalled in modern history (Giovannucci and Purcell, 2008). Thus, agriculture department in many countries should have a proper channel for plants or crops to go through the trading process. The spread of GY in Asia, as well as for many other diseases, can be checked and controlled by rigorous pest monitoring and trading of certified plants.

## Author Contributions

RP, AL and HKG conceived the review; RP, TS, HG and MV analyzed the literature data; TS prepared the maps; RP, HG, MV and HKG prepared the manuscript; AL, LB and HKG edited the manuscript.

## Conflict of Interest Statement

The authors declare that the research was conducted in the absence of any commercial or financial relationships that could be construed as a potential conflict of interest.
